# Daily self-efficacy, planning and social support explain leisure-time physical activity in working adults: evidence for the cultivation hypothesis from an ambulatory assessment study

**DOI:** 10.1080/21642850.2025.2576610

**Published:** 2025-11-12

**Authors:** Lea O. Wilhelm, Nina Knoll, Lotte-Eleonora Diering, Karolina Kolodziejczak-Krupp, Jana Maas, Hendrik Schmidt, Lena Fleig

**Affiliations:** a Department of Psychology, MSB Medical School Berlin, Berlin, Germany; b Department of Education and Psychology, Freie Universität Berlin, Berlin, Germany; c Institute of Medical Sociology, Charité – Universitätsmedizin Berlin, Berlin, Germany; d Day Clinic Manual Medicine, Sana Hospital Lichtenberg, Berlin, Germany; e Julius-Wolff-Institut, Berlin Institute of Health, Charité Universitätsmedizin Berlin, Berlin, Germany

**Keywords:** Physical activity, accelerometer, social support, workplace, mediation

## Abstract

**Background:**

Identifying theory-based, modifiable processes and resources in everyday life is essential for improving physical activity levels, and leisure-time physical activity (LTPA) especially. Deconfounding different sources of social support for day-to-day LTPA, we examined effects of LTPA-specific received support from both private and work domains. Furthermore, we investigated whether daily intrapersonal processes – such as positive affect, self-efficacy, and planning – are linked with LTPA through cultivating social support.

**Methods:**

A total of 118 adults (*M*_age_ = 37.88, *SD *= 11.73, 65% women) participated in a 15-day ambulatory assessment study. Morning positive affect, self-efficacy, and planning, afternoon LTPA-specific received social support from family/friends, and colleagues/supervisors, and self-reported LTPA and working hours (both measured in the evening) were assessed daily. LTPA was also measured using accelerometers combined with worktime information. First, we fit multilevel models to explain device-assessed and self-reported LTPA. Second, within-person mediation analyses examined the role of received social support as a potential mediator between relevant intrapersonal processes and LTPA at the day level.

**Results:**

On average, participants received more LTPA-specific support from the private compared to the work domain. Having received higher-than-usual social support from family/friends on a day was consistently linked to higher LTPA. No such within-person effect emerged for support from colleagues/supervisors. On days with higher-than-usual self-efficacy and planning, participants performed more LTPA, whereas positive affect was unrelated to LTPA at the within-person level. These results were found for both device-assessed and self-reported LTPA. Within-person mediation analyses revealed that between 16%−22% of the effects from self-efficacy and planning to device-assessed/self-reported LTPA were mediated by social support from family/friends.

**Conclusions:**

Our results identify intrapersonal and private-domain social exchange processes as potential targets for future ecological momentary interventions. Consistent with the cultivation hypothesis, we also identified that daily self-efficacy and planning were linked to LTPA via social support from family/friends.

## Background

Regular physical activity (PA) benefits both physical and mental health, and has been associated with reduced risks of conditions like depression, cardiovascular diseases, and certain cancers (Ahmadi et al., 2022; Hemmingsson et al., 2022; Pearce et al., 2022; You et al., 2025). However, an estimated 28% of adults worldwide do not meet World Health Organization (WHO)-recommended PA guidelines (Guthold et al., 2018). Leisure-time physical activity (LTPA), which includes any non-work-related PA such as cycling or housework, offers distinct health benefits, compared to occupational PA (Hallman et al., 2017). Given that adults spend a considerable portion of their waking hours working, their LTPA may be constrained not only by limited time but also by workplace stressors and resources that can either hinder or facilitate LTPA. The present paper aims to explore how social relationships from the private and work domains support LTPA engagement and how intrapersonal processes, specifically positive affect, self-efficacy and planning, may cultivate the receipt of LTPA-specific support.

### Social support for LTPA

Social relationships play an important role in shaping individual health behaviours, particularly through social support. Social support is an interpersonal mechanism that involves the exchange of resources (Cohen et al., 2000). Received social support is assessed retrospectively and refers to past support exchanges (Knoll et al., 2018). Support may have different functions, i.e., instrumental (e.g., tangible help), emotional (e.g., consolation), or informational (e.g., advice), although these functions are often closely related empirically (e.g., Murray et al., 2023). However, evidence linking social support and PA remains inconsistent (Scarapicchia et al., 2017), and may vary depending on the source of support (Sarkar et al., 2016). This study investigates differential effects of daily received support from the workplace (colleagues/supervisors) and private life (family/friends) on LTPA.

### Theory-based intrapersonal processes involved in LTPA

**Figure 1.**
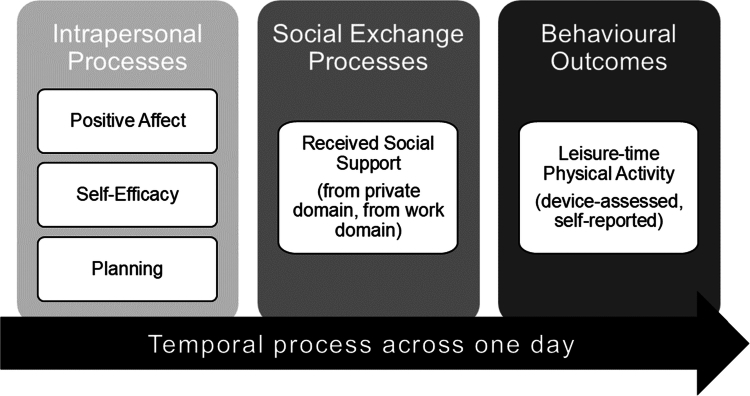
Theory-based intrapersonal processes and social exchange processes in the present study.

The ways in which social exchange processes work together with intrapersonal processes contributing to behaviour change remain poorly understood (Rothman et al., 2020). As illustrated in Figure 1, we examined three theory-based intrapersonal processes (i.e., positive affect, self-efficacy, planning) with well-established links to PA (Buecker et al., 2021; Carraro & Gaudreau, 2013; Zhang et al., 2019) to investigate their potential to foster social support. Health behaviour change theories (e.g., Health Action Process Approach; HAPA; Schwarzer, 2008) emphasize the role of self-efficacy, the belief in one's capability to perform a behaviour despite obstacles. Another relevant process is planning, a mental self-regulatory strategy, that aids translating intentions into behaviour (Schwarzer, 2008). Increasingly, scholars have suggested incorporating implicit processes, such as positive affect, into dual-process models for health behaviour (Hofmann et al., 2008; Strobach et al., 2020). High positive affect (such as joy) may enhance momentary readiness to engage in PA by expanding a person's thought-action repertoire (Fredrickson, 1998). This study investigated whether daily fluctuations in positive affect, self-efficacy, and planning, facilitate LTPA by cultivating social support from others.

### Interplay between intrapersonal processes and social support

According to the *cultivation hypothesis,* intrapersonal processes such as self-efficacy help persons to elicit social support (Schwarzer & Knoll, 2007). The likelihood of receiving social support depends on situational, provider, as well as recipient characteristics, such as demonstrating active coping (Dunkel-Schetter & Skokan, 1990; Iida et al., 2008; Schwarzer & Weiner, 1991). Individuals who display high levels of self-efficacy and planning may be perceived as actively coping, thereby attracting social support from others. Self-efficacy could also encourage individuals to engage in social activities, thus providing an opportunity to receive social support (Schwarzer & Knoll, 2007). Positive affect may also increase the likelihood of receiving support. Research from the provider's perspective (Carlson et al., 1988) suggests that support providers in a good mood were more likely to respond with prosocial behaviour towards strangers. In a similar way, recipient positive affect may spill over from recipient to provider, increasing the chance for support (Telle & Pfister, 2016). However, prior dyadic studies did not confirm that recipients' positive affect is associated with support provision (Iida et al., 2008; Knoll et al., 2011). Hence, the role of positive affect in promoting LTPA via social support warrants further investigation.

Based on theory and previous research (Banik et al., 2021; Hohl et al., 2016; Knoll et al., 2011; Schwaninger et al., 2021), we examined the cultivation hypothesis in an intensive-longitudinal design, and assessed whether daily associations of positive affect, self-efficacy, and planning, with LTPA, are mediated by social support (Figure 1).

### Temporal dynamics in intrapersonal and social exchange processes

To capture these processes in daily life, we used ambulatory assessment (AA). Although theories often assume within-person dynamics, past research has frequently investigated between-person differences. Studying processes at the within-person level can provide critical insights into underlying dynamics of theory (Hamaker, 2025). Findings may be different at the between-person level (Bierbauer et al., 2017; Kolodziejczak-Krupp et al., 2025). Prior AA studies demonstrated positive within-person level associations between PA and positive affect, self-efficacy, and planning (Anderson, 2021; Niermann et al., 2016; Pickering et al., 2016). Although prior studies provide promising evidence of positive associations between daily social support and PA, most have focused on romantic relationships and all-day PA rather than LTPA (Berli et al., 2018; Khan et al., 2013; Martire et al., 2013). This study therefore examined the day-level predictive power of intraindividual processes (self-efficacy, planning and positive affect) and social support to understand their practical role in LTPA. While evidence for single paths of the cultivation hypothesis exists (Schwaninger et al., 2021), no previous work has examined the cultivation hypothesis as a within-person mediation process on a daily timescale.

### Aims and hypotheses

Our study aimed to examine how receiving social support from two domains – private (family/friends) and work (colleagues/supervisor) – relates to LTPA among working adults in their daily lives. While prior studies mostly relied on LTPA self-report measures, our study design allowed for a precise assessment of device-assessed LTPA, further validating results with self-reported LTPA. We also examined the role of time-varying intrapersonal processes (positive affect, self-efficacy, and planning) in relation to daily LTPA. To explore the interplay of intrapersonal and social exchange processes in daily life, we tested whether social support serves as a mediator between intrapersonal processes and LTPA at the within-person level (*cultivation hypothesis*).

We hypothesized that higher-than-usual social support from family/friends (H1a) or colleagues/supervisors (H1b) on a given day are associated with more LTPA than usual. Higher-than-usual positive affect (H2a), self-efficacy (H2b), and planning (H2c) on a day are associated with more LTPA than usual. The positive associations between daily intrapersonal processes (positive affect, self-efficacy, planning) and LTPA on a day are mediated by higher-than-usual social support from family/friends (H3a) and colleagues/supervisors (H3b). Additionally, we explored the between-person level associations of social support, positive affect, self-efficacy, and planning, with LTPA.

## Method

### Study design

This study investigated the primary outcome of LTPA with AA data from the workHealth project, an observational, prospective-longitudinal study with working adults from the general population across two months, with an intensive-longitudinal design phase of 15 days. The present study does not focus on the prospective-longitudinal follow-up online questionnaires conducted at two, four, and eight weeks. More detailed information on the study design can be found in our study protocol (Wilhelm et al., 2025).

### Participants

Individuals were eligible if they were working either in a sedentary occupational setting (e.g., office work) or as physiotherapists, and at least part-time (i.e., 20 hours/week). Participants had to be between 18 and 64 years old, be able to hear an alarm on the smartphone and read and understand the German study materials. Detailed exclusion criteria are described in Wilhelm et al. (2025).

Of 144 participants starting the study, two participants dropped out of the study during the AA phase (reasons: technical difficulties, illness). Participants were excluded from analyses for two reasons: (i) they had less than three valid days for the accelerometer data (*n* = 8, including two dropouts), or (ii) they worked less than 20 hours/week (*n* = 16). Figure S1 (Supplementary Material S1) depicts a data availability flowchart. Participants in the final sample (*n* = 118) were on average 37.88 years old (*SD* = 11.73, range = 19−65), and 65% were women. Forty-seven (40%) were physiotherapists, and 71 (60%) were from a sedentary occupational context. Table 1 displays detailed descriptive information for both the final and excluded participant sample.

**Table 1.** Demographic information for participants in final sample (*n* = 118) and dropout/excluded participants (*n* = 26) with difference tests.

Variable	Final sample (*n* = 118)		Dropout/excluded sample (*n* = 26)		Difference test			
	*M/n*	*SD/%*	*M/n*	*SD/%*	χ²/*t*	*df*	*p*	*Cohen's d/Cramer's V*
Age	37.88	11.73	24.50	4.66	−9.46	101.37	**<.001**	−1.24
Gender: man^a^	41	34.75	6	23.08	0.84	1	.359	0.10
Body mass index (kg/m²)	23.63	2.88	21.98	2.71	−2.77	38.43	**.009**	−0.58
Occupational context: sedentary^b^	71	60.17	23	85.46	6.33	1	**.012**	0.24
Working hours	34.74	8.07	15.56	14.81	9.45	33.50	**<.001**	−2.39
Primarily working from home	17	14.41	8	30.77	3.96	1	**.014**	0.24
Students	15	12.71	21	80.77	49.06	1	**<.001**	0.60
Mother tongue: German^c^	106	89.83	25	96.15	0.41	1	.522	0.08
Marital status								
Married	35	29.66	1	3.85	9.47	3	**.024**	0.26
In committed relationship	50	42.37	16	61.54				
Divorced	5	4.24	0	0				
Single	28	23.73	9	34.62				
Having children	45	38.14	2	7.69	7.65	1	.006	0.25
Living situation								
With partner	68	57.63	6	23.08	8.85	1	**.003**	0.27
With parent/s	6	5.08	5	19.23	4.20	1	**.040**	0.20
With children	29	24.58	2	7.69	2.67	1	.103	0.16
With others	14	11.86	6	23.08	1.40	1	.237	0.12
Alone	29	24.58	11	40.74	1.44	1	.231	0.12
Highschool diploma	99	83.90	26	100	3.73	1	.053	0.18
Low income^d^	12	10.17	22	84.62	61.41	1	**<.001**	0.67
Smoker	19	16.10	6	23.08	0.32	1	.573	0.07

Note: All listed demographic characteristics were self-reported at baseline. No data on ethnicity were collected. Significant *p*-values (*p* < .05) are printed in bold.

^a^ reference category: Woman (zero responses for other gender identifications).

^b^ reference category: Manual occupational context (physiotherapist).

^c^ reference category: Mother tongue not German.

^d^ reference category: Income not low. Low income is operationalized as a household income of <1250€.

### Procedure

For study recruitment we used various communication channels including flyers and online forums like Twitter. When potential participants made contact, participation information was sent via email, and eligibility criteria were checked by telephone. During a baseline session, participants gave informed written consent, and completed a baseline questionnaire, followed by a spinal shape and mobility assessment, which is not part of the current study. Participants then received two devices, an accelerometer (Move4, movisens GmbH, Germany) and a study smartphone equipped with the app movisensXS (movisens GmbH, Germany), and were trained how to use them. During the intensive-longitudinal design phase of 15 days, participants were invited to fill out three daily questionnaires on their study smartphone prompted at 8 am, 3 pm, and 9 pm (i.e., ecological momentary assessment [EMA] surveys). Participants wore the accelerometer during waking hours on the right hip.

### Measures

#### LTPA

Our primary outcome was LTPA in minutes per day. We assessed LTPA of moderate-to-vigorous intensity (MVPA) device-based with the Move4 accelerometer (data segmented by self-reported working hours), and as a self-report in the end-of-day EMA survey. Detailed device-assessed LTPA measure descriptions are reported in our study protocol (Wilhelm et al., 2025). Move4 accelerometer raw data were pre-processed with the software DataAnalyzer (movisens GmbH, Germany), to determine minute-by-minute wear-time information and MVPA (accounting for age, height, weight, and gender). We combined this information with working hours, self-reported in the evenings with the item ‘Did you work today?’. If participants had worked, the question ‘From when until when did you work today?’ was presented, to be answered by specific times (e.g., 9.15 am–4 pm). We also assessed timing for one longer break of at least 15 minutes. If worktime information was missing in the evening EMA survey, it could be inferred from the afternoon EMA survey item ‘Are you still working?’ (response options: 1 = *yes*; 2 = *no*; 0 = *I did not work today*). If participants selected response option 0 (*I did not work today*), the entire day was coded as leisure-time. For response options 1 (*yes*) and 2 (*no*), no replacement of missing worktime was made, since these responses indicated a working day but did not provide information on exact working hours. We then aggregated MVPA-minutes, across non-work minutes when the accelerometer had been worn, to create a measure of device-assessed LTPA. We aggregated all-day accelerometer-wear-time, and leisure-time-specific wear-time across days. We excluded days when the accelerometer had been worn for less than 10 hours all-day (Troiano et al., 2008; You, 2024). Due to our leisure-time focus, we also excluded days when the accelerometer had not at all been worn during leisure-time. All-day accelerometer wear-time >10 h and leisure-time-specific wear-time >0 h, as classified by our procedure described above, constituted a ‘valid wear-day’. We excluded participants with less than three valid wear-days (Matthews et al., 2002; You et al., 2024).

Self-reported daily LTPA (not limited to MVPA) was assessed in the evening EMA survey. Participants were asked ‘Have you been physically active during your leisure-time today?’ and if they indicated 1 (*yes*), kind of activity, start time, and duration of the activity in minutes were assessed. Participants could report up to three different activities for the day. Duration in minutes were summed across all three activities to indicate daily self-reported LTPA. We excluded days of self-reported LTPA with durations above 16 hours, minus working hours.

#### Social support from family/friends and from colleagues/supervisor

LTPA-specific received social support from family/friends and from colleagues/supervisors was assessed in the afternoon EMA survey with two single items adapted from Scholz et al. (2016), referring to two domains of life. The item stem ‘Today, I received support from…’ was followed by ‘…friends or family…’ to indicate *social support from family/friends* (private domain), and ‘…colleagues or supervisor…’ to indicate *social support from colleagues/supervisors* (work domain), ending with ‘…with regard to my leisure-time physical activity’. Participants answered the items on a 6-point Likert scale from 1 (*not at all true*) to 6 (*absolutely true*). We correlated 15-day-averaged single-item scales with multi-item scales (Wilhelm et al., 2025) from follow-up questionnaires at two weeks to demonstrate concurrent validity, and found strong correlations (social support from family/friends, *r* = . 54, *p* < .001; social support from colleagues/supervisors, *r* = .53, *p* < .001, respectively).

#### Positive affect

Momentary positive affect was assessed in the morning EMA survey with an adapted short form of the Positive and Negative Affect Schedule (Knoll et al., 2020; Mackinnon et al., 1999; Watson et al., 1988). Five positive affect items (*alert, inspired, enthusiastic, determined, excited)* were averaged to create a mean score. Responses were provided on a 5-point Likert scale from 1 (*very slightly or not at all*) to 5 (*extremely*). The between-person reliability across days was R_kF_ = .98. The within-person reliability of day-to-day changes was R_change_ = .77 (Cranford et al., 2006).

#### Self-efficacy and planning

LTPA-specific self-efficacy and action planning were assessed in the morning EMA survey with a single item each, adapted from Sniehotta, Scholz et al. (2005), and Sniehotta, Schwarzer et al. (2005), respectively. Self-efficacy was assessed with ‘I am confident that today I can manage to be physically active in my leisure-time, even if it is difficult’. Planning was assessed with ‘For today, I have already made a detailed plan on which occasions I can be physically active during my leisure-time’. Responses for these variables were provided on a 6-point Likert scale from 1 (*not at all true*) to 6 (*absolutely true*). Our 15-day-averaged single-item scales correlated strongly with follow-up questionnaires at two weeks (self-efficacy, *r* = .53, *p* < .001; planning, *r* = .62, *p* < .001, respectively), demonstrating concurrent validity.

### Data analysis

#### Descriptive analyses

Data were analysed in R (version 4.4.1), and MPlus (Version 8.7). Due to technical issues, two EMA survey responses had been given outside the 3-hour window. They were set to missing. Univariate plausible outliers on device-assessed and self-reported LTPA were 90th percentile-winsorized to reduce their impact (Aguinis et al., 2013). We evaluated dropout mechanisms by calculating differences between the final sample and dropout/excluded participants with *t*-tests and χ²-tests. Dependent *t-*tests were calculated to compare frequencies of social support from family/friends vs. from colleagues/supervisors. Correlations at the between-person level were computed with Pearson's correlations, and at the within-person level with repeated-measures correlations (Bakdash & Marusich, 2017), respectively. An alpha-level of .05 was considered significant. An a-priori sample size planning was based on heuristics from Arend and Schäfer (2019), indicating a minimum of 150 participants for examining within-person associations.

#### Multilevel models for associations with LTPA

Time-varying predictors were group-mean centered using grand-mean centered raw scores (Bolger & Laurenceau, 2013; Vuorre & Bolger, 2018). This created person-average deviations from the sample (between-person predictors) and day-to-day fluctuations from the person mean (within-person predictors). Time was centered on day 1 (0 = day 1). Device-wear-time and age were grand-mean centered. Two-level models with days (Level-1) nested in participants (Level-2) were used to determine same-day associations between predictors and device-measured LTPA, accounting for covariates. A first model (Model A) included social support from family/friends and from colleagues/supervisors as within- and between-person level predictors. A second model (Model B) included social support from family/friends, positive affect, self-efficacy, and planning as within- and between-person level predictors. We controlled for a linear time trend, work day, leisure-time device-wear-time, weekend, and between-person level covariates age, gender, occupational context (0 = physiotherapists; 1 = sedentary), and being single as fixed effects. Missing data were excluded listwise. We estimated models using restricted maximum likelihood with the R package *nlme* (Pinheiro et al., 2025). Where possible, a maximal random effects structure was specified (Barr et al., 2013). When encountering convergence issues, we reduced random effects until model convergence, giving priority to social support, positive affect, self-efficacy and planning. Based on model comparisons, we chose a covariance structure of independent error terms. We conducted sensitivity analyses controlling for all characteristics on which the initial and the final sample differed, to assess the robustness of our findings. Analyses were re-run with self-reported LTPA as outcome. Due to zero-inflated values, we estimated negative binomial generalized linear mixed models using the *glmmTMB* package (Brooks et al., 2017). With the exception of leisure-time device-wear-time (omitted), time-varying predictors and covariates were identical to analyses with device-assessed LTPA as outcome.

#### Within-person mediation analyses for LTPA

**Figure 2.**
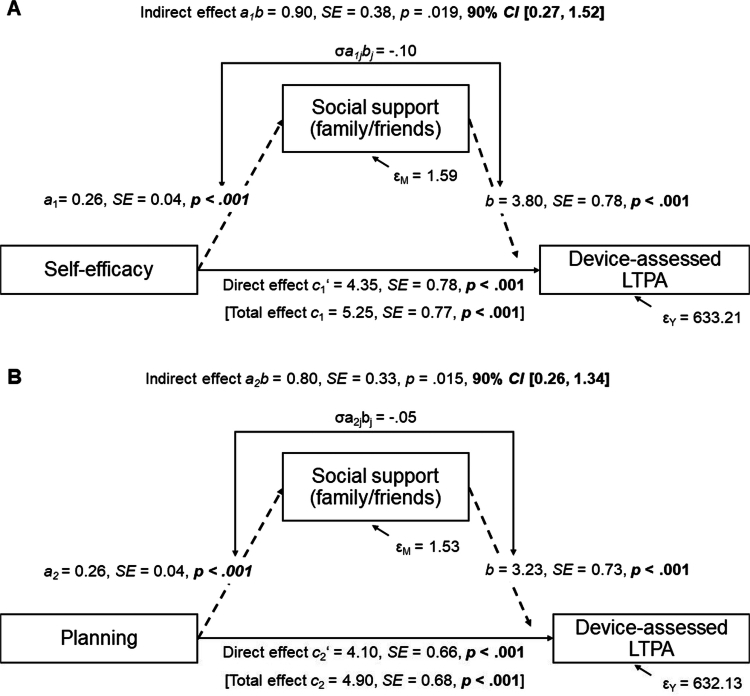
Within-person mediation analyses results, separately for self-efficacy (A), and for planning (B) as independent variables. Note: Within-person mediation analysis of intrapersonal processes LTPA-specific self-efficacy (A) or LTPA-specific planning (B), the mediator LTPA-specific received social support (family/friends, M), and the dependent variable device-assessed LTPA (Y). Covariates (Level-1): linear time trend, working day, weekend, leisure-time device-wear-time. Dotted lines show indirect effects a_1_b (A), or a_2_b (B). The subscript j signifies the random slope for participants. 1120 observations, 108 participants (due to missings, or lack of within-person variation). SE = Standard Error; CI = Confidence Intervals; LTPA = Leisure-time physical activity; M = Mediator; Y = Dependent variable.

Within-person predictors were used in a within-person mediation analysis (1−1−1 mediation) in MPlus. Between-person level mediation was not examined in the present study. We examined social support from family/friends as a daily mediator (M) of the link between self-efficacy (independent variable 1, X1), and planning (independent variable 2, X2), and the dependent variable device-assessed LTPA (Y). For reasons of collinearity of the proposed independent variables (Hayes, 2013, p. 154), two separate mediations models were fit (Figure 2). Due to non-significance of within-person level social support from colleagues/supervisors, and within-person level positive affect, we did not test them as mediator or independent variable, respectively. We excluded 10 participants who showed no variation on social support. Following Bolger and Laurenceau (2013), we used within-person-centered X1, X2, M, and Y variables, and a full-information maximum likelihood estimation. Referring to previous intensive-longitudinal design literature using within-person mediation analysis (Berli et al., 2018; Martin et al., 2021), M was regressed on X1 (*a*1 path), or on X2 (*a*2 path). We regressed Y on M (*b* path) and X1, or X2 (*c*1′/*c*2′ path). The total effects *c* (*c*1/*c*2) encompass the direct effects *c*′ (*c*1′/*c*2′), the product of *a (a*1*/a*2) and *b* effects, and the covariance σ_*ajbj*_ (σ_*a*1j*bj*_/σ_*a*2j*bj*_) between *a (a*1*/a*2) and *b* effects (Bolger & Laurenceau, 2013). An average indirect effect was calculated as the *ab (a*1b/*a*2b) product, and the covariance σ_*ajbj*_ (σ_*a*1j*bj*_/σ_*a*2j*bj*_). Because of our directional, one-sided hypotheses, we tested 90% confidence intervals (CI) for the indirect effect. The mediation models controlled for a linear time trend, work day, leisure-time device-wear-time, and weekend. Analyses were re-run with self-reported LTPA as dependent variable.

### Ethics statement

This study received ethical approval from the Medical School Berlin's ethics committee on 29 March 2021 (approval number: MSB−2021/62). Pre-registration took place on 28 June 2021 in the German Clinical Trials Register (DRKS-ID: DRKS00025296). The study ran from 2021 to 2024. Reimbursement for study participation was a minimum of 40 euros, and course credits, where applicable.

## Results

### Compliance rate and dropout analysis

The overall compliance rate for EMA survey prompts for the initial sample of *N* = 144 was 88%. A minimum of three valid days of accelerometer data was provided by 93% of participants. Participants responded to the EMA surveys with a latency of *M*(*SD*) = 33.07(33.11, range = 0−170) minutes. Participants rated their days as moderately typical, *M*(*SD*) = 3.67(1.47, range = 1−6). Participants who were excluded from analyses (*n* = 27, data availability flowchart in Supplementary Material S1), including two dropout participants, were similar to participants in the final analysis sample (*n* = 118; see Table 1 for socio-demographic information and difference test statistics).

### Descriptive analyses for daily variables

Table 2 displays descriptive statistics and correlations of the variables under study. Intra-class correlation coefficients (ICCs) indicated that between-person differences accounted for 20%−31% of the variance in the daily variables. Only positive affect displayed higher variability due to between-person differences with 45%. There were positive, medium-to-strong associations of device-assessed with self-reported LTPA (*r*_between-person_ = .47, *p* < .001; *r*_within-person_ = .50, *p* < .001). Participants received higher levels of support from family/friends than from colleagues/supervisors, *t*(117) = 8.86, *p* < .001, *d* = 0.82.

**Table 2.** Means, standard deviations, intra-class correlation coefficients and between-person level and within-person level correlations between daily variables.

Variable	*M*	*SD*	Range	% missing	Correlations, ICCs						
					1.	2.	3.	4.	5.	6.	7.
1. Device-assessed LTPA (leisure-time MVPA/day)	58.07	37.85	8−147	20.28	(.31)	.50***	.04	.27***	.27***	.02	.33***
2. Self-reported LTPA (leisure-time PA minutes/day)	41.47	52.83	0−180	9.72	.47***	(.20)	.04	.38***	.40***	.07**	.37***
3. Positive affect	2.32	0.81	1−4.6	8.87	.12	.24**	(.45)	.12***	.10***	.03	.01
4. Self-efficacy	3.64	1.70	1−6	9.27	.32***	.48***	.21*	(.30)	.74***	.16***	.28***
5. Planning	3.21	1.90	1−6	9.27	.40***	.58***	.35***	.76***	(.24)	.16***	.30***
6. Social support from colleagues/supervisors	1.48	1.11	1−6	9.77	.26**	.05	.05	.07	.15	(.27)	.18***
7. Social support from family/friends	2.26	1.69	1−6	9.77	.20*	.36***	.11	.30**	.25**	.42***	(.31)

Note: 1307 ≤ *n* ≤ 1613 observations (due to missings), 118 participants. *M *= Mean; *SD* = Standard deviation; ICCs = Intra-class Correlation Coefficients; LTPA = leisure-time physical activity; MVPA = moderate-to-vigorous physical activity; PA = Physical activity. Correlations at the within-person level (repeated measures correlations; Bakdash & Marusich, 2017) are reported above the diagonal, correlations at the between-person level (Pearson correlations) between aggregated person-means are reported below the diagonal. ICCs are reported in the diagonal. Correlation coefficients with significant *p*-values (*p* < .05) are printed in bold. *p < .05; **p < .01; ***p < .001.

### Hypothesis tests: associations between social support and LTPA

In a first step, we analysed within-person and between-person associations of social support from family/friends and from colleagues/supervisors with device-assessed LTPA (Model A, Table 3) in a multilevel model. As hypothesized (H1a), at the *within-person level,* we found that on days with higher support from family/friends than usual, participants performed more device-assessed LTPA. For a 1-unit increase in social support from family/friends, an average participant engaged in 5.74 MVPA-minutes of device-assessed LTPA more, other variables held constant. In contrast to H1b, we found no evidence for an association with within-person level social support from colleagues/supervisors. At the *between-person level*, social support from colleagues/supervisors, but not from family/friends was related to device-assessed LTPA: The more overall LTPA-related social support participants received from the work domain, the more physically active they were during leisure-time. Compared to a random-intercept-only model with fixed effect covariates, Model A with social support from two sources explained 5% more unexplained variance in the outcome.

**Table 3.** Parameter estimates from mixed model testing daily social support from colleagues/supervisors and from family/friends (Model A) and daily social support from family/friends, positive affect, self-efficacy, and planning (Model B) and their link with daily leisure-time physical activity (device-assessed MVPA during leisure-time/day, winsorized 90th percentile).

	Model A			Model B		
Fixed effects	**B* (*SE*)*	95% CI	*p*	**B* (*SE*)*	95% CI	*p*
Intercept	59.59 (4.45)	50.86–68.32	**<.001**	57.25 (4.31)	48.80–65.70	**<.001**
Linear time^a^ (reference = day 1)	−0.03 (0.18)	−0.39–0.32	.851	−0.04 (0.18)	−0.39–0.31	.826
Working day	−1.95 (3.16)	−8.14–4.25	.538	−0.50 (3.10)	−6.58–5.57	.871
Wear-time during leisure (in hours)	3.52 (0.37)	2.79–4.25	**<.001**	3.64 (0.37)	2.92–4.36	**<.001**
Weekend	−6.90 (2.52)	−11.84 to −1.97	**.006**	−4.75 (2.49)	−9.63–0.13	.057
*Within-person effects*						
Social support from colleagues/supervisors	0.27 (1.10)	−1.89–2.43	.808			
Social support from family/friends	5.74 (0.75)	4.26–7.21	**<.001**	4.07 (0.70)	2.70–5.44	**<.001**
Positive affect				3.07 (1.63)	−0.14–6.27	.061
Self-efficacy				2.01 (0.92)	0.21–3.81	**.028**
Planning				2.62 (0.77)	1.11–4.13	**.001**
*Between-person effects*						
Social support from colleagues/supervisors	8.21 (3.07)	2.14–14.29	**.009**			
Social support from family/friends	0.96 (2.09)	−3.18–5.10	.647	−0.39 (1.85)	−4.06–3.29	.835
Positive affect				0.17 (3.32)	−6.42–6.76	.958
Self-efficacy				−2.04 (2.76)	−7.51–3.43	.461
Planning				9.61 (2.75)	4.16–15.05	**.001**
Age	0.11 (0.18)	−0.24–0.47	.522	−0.04 (0.17)	−0.38–0.29	.793
Gender: man (reference = woman)	2.97 (4.02)	−5.00–10.94	.461	1.49 (3.91)	−6.26–9.24	.704
Occupational context: sedentary (reference = physiotherapist)	−3.50 (4.18)	−11.79–4.79	.405	−0.40 (4.02)	−8.36–7.56	.920
Being single	10.01 (4.51)	1.07–18.94	**.028**	8.57 (4.26)	0.13–17.02	**.047**

Random effects ([co-]variances)	Var	95% CI		Var	95% CI	
*Level−2 (between-person)*						
Intercept	369.63	270.53–505.03		355.78	258.22–490.19	
*Within-person effects*						
Social support from colleagues/supervisors	21.05	7.03–63.10				
Social support from friends/family	15.45	6.65–35.88		8.90	2.96–26.78	
Positive affect				73.28	27.05–198.55	
Self-efficacy				11.31	1.59–80.61	
Planning				7.90	1.27–49.16	
Intercept and support from colleagues/supervisors	−0.06	−0.71–0.64				
Intercept and support from friends/family	0.57	0.07–0.84		0.74	0.01–0.96	
Intercept and positive affect				−0.37	−0.71–0.11	
Intercept and self-efficacy				0.23	−0.36–0.68	
Intercept and planning				0.21	−0.29–0.62	
Support from colleagues/supervisors and from friends/family	−0.78	−0.99–0.44				
Support from friends/family and positive affect				−0.44	−0.93–0.62	
Support from friends/family and self-efficacy				−0.40	−0.91–0.60	
Support from friends/family and planning				0.73	−0.81–0.99	
Positive affect and self-efficacy				0.45	−0.53–0.92	
Positive affect and planning				−0.08	−0.48–0.35	
Self-efficacy and planning				−0.67	−0.97–0.49	
*Level−1 (within-person)*						
Residuals	704.69	647.68–766.72		613.18	557.63–674.26	
Model fit						
Deviance	12446.43			11520.87		
AIC/BIC	12456.03/12559.40			11547.63/11715.52		
*R*² (marginal/conditional)	.22/.51			.27/.57		

Note: 1298 observations, 118 individuals (Model A), 1214 observations, 118 individuals (Model B). LTPA = leisure-time physical activity, here: moderate-to-vigorous intensity; MVPA = moderate-to-vigorous physical activity; SE = Standard Error; CI = confidence interval with lower level and upper level; AIC = Akaike information criterion; BIC = Bayesian information criterion. Unstandardized coefficients are displayed. Significant *p*-values (*p* < .05) are printed in bold. Due to model non-convergence, no random effects were specified for linear time.

*Within-person level* effects for social support from two domains for self-reported LTPA followed the same pattern (Supplementary Material S2). *Between-person level* effects differed: Social support from colleagues/supervisors was unrelated to self-reported LTPA. Social support from family/friends was significantly and positively linked to self-reported LTPA.

Sensitivity analyses for analyses controlling for all identified differences between the final sample and excluded participants showed the same result pattern (Supplementary Material S3).

### Hypothesis tests: associations of positive affect, self-efficacy, and planning with LTPA

In a second step, we estimated a model explaining device-assessed LTPA by within-person and between-person level social support from family/friends, positive affect, self-efficacy, and planning (Model B, Table 3). We did not include the non-significant proposed mediator social support from colleagues/supervisors. In line with H2b and H2c, at the *within-person level*, higher-than-usual self-efficacy and planning on a day were related to more device-assessed LTPA that day. Contrary to H2a, within-person positive affect was unrelated to device-assessed LTPA. At the *between-person level*, planning, but not positive affect or self-efficacy, was related to device-assessed LTPA: On average, the more participants planned in the morning, the higher was their device-assessed LTPA. Compared to a random-intercept-random-slope model with fixed and random effects for social support from family/friends, and covariates, Model B explained 7% more variance in the outcome.

At the *within-person level,* result patterns for positive affect, self-efficacy, and planning were the same for self-reported LTPA as outcome (Supplementary Material S2), confirming H2b and H2c, but not H2a. *Between-person level* result patterns were mostly the same, but we found an additional link from self-efficacy to self-reported LTPA.

A sensitivity analysis controlling for all identified differences between the excluded participants and the final sample showed the same result pattern (Supplementary Material S3).

### Hypotheses tests: within-person mediation analyses for LTPA

We analysed at the within-person level whether social support from family/friends mediated the effect from self-efficacy and planning on device-assessed LTPA. Since social support from colleagues/supervisors and positive affect did not emerge as relevant within-person processes related to LTPA, we did not run further mediation analyses for these variables. Also, no lagged effects were considered.

Results from within-person mediation analyses are displayed in Figure 2 (see also Supplementary Material S4). Using separate mediation models for self-efficacy and planning as independent variables, we found evidence for an indirect effect from self-efficacy (Figure 2A), and planning (Figure 2B) via social support from family/friends to device-assessed LTPA. The indirect effects accounted for 17%/16% of the total effects from self-efficacy, and planning, to device-assessed LTPA, respectively. Thus, findings confirmed H3a, that social support from family/friends was a within-person mediator in the daily link between self-efficacy, and planning, and LTPA. For self-reported LTPA as dependent variable the same result patterns emerged (Supplementary Material S4). Indirect effects accounted for 20/22% of the total effects.

## Discussion

This study investigated day-to-day fluctuations in social support from two domains (work and private domain) and key intrapersonal processes in relation to LTPA among working adults. Consistent with our hypotheses, at the within-person level, social support from family/friends was positively associated with daily LTPA. In contrast to hypotheses, no association emerged for within-person level social support from colleagues/supervisors. Regarding theory-based, intrapersonal processes, our findings revealed that self-efficacy and planning operate as dynamic, daily processes that are related to higher levels of LTPA, beyond the effects of social support from family/friends. Contrary to expectations, positive affect did not emerge as within-person predictor of LTPA. Our within-person mediation analysis results support that social support from family/friends partially mediates the daily association between LTPA and self-efficacy and planning, respectively. At the within-person level, consistent patterns of results were found for device-assessed and self-reported PA.

### Social support for LTPA

Our study supports that social support in the private domain serves as a daily, within-person level resource for LTPA, as evidenced by both device-assessed and self-report measures. This finding is in line with prior research with romantic couples and all-day PA (Berli et al., 2018; Khan et al., 2013; Schwaninger et al., 2021). However, contrary to our expectations, we did not find a within-person link between LTPA and social support from the work domain. This may reflect greater interdependence and dynamic coordination of LTPA goals with family and friends, compared to workplace relationships (Carr et al., 2019; Fitzsimons et al., 2015). Colleagues and supervisors may be less motivated to provide support for a leisure-time goal, less aware of non-work-related demands, and more restricted in their possibilities to provide support due to workplace demands, making need-contingent LTPA-support in a timely manner less likely. Finally, norms and expectations around support provision related to health behaviour may differ between workplace and private settings.

At the between-person level, our study found differing results between device-assessed and self-reported LTPA in relation to social support from the two domains. In line with the within-person findings, only support from family/friends (not from colleagues/supervisors), emerged as significant predictor of self-reported LTPA. For device-assessed LTPA, however, average between-person level social support from family/friends was not a significant predictor, whereas social support from colleagues/supervisors was. These findings mirror overall inconclusive and heterogeneous evidence of between-person associations of received support with PA from individual-based and couples-based observational studies, and meta-analyses (e.g., Berli et al., 2018; Carr et al., 2019). The between-person level finding for device-assessed LTPA aligns with prior studies highlighting that people, on average are more physically active in their leisure-time if they receive social support from the workplace (Sarkar et al., 2016). Flexible and timely support provision by colleagues and supervisors could be constrained by work-related demands so that daily fluctuations in support from workplace settings may not translate to higher LTPA. Instead, high overall levels of LTPA-specific support from the workplace may coincide with a more conducive overall organizational health climate (Sonnentag & Pundt, 2016) and a health-promoting workplace culture, thereby promoting long-term employee engagement in LTPA.

### Intrapersonal processes involved in LTPA

Our findings confirm the hypothesized same-day, within-person effects of self-efficacy and planning on LTPA, consistent across both device-assessed and self-reported LTPA measures. At the between-person level, self-efficacy and planning were also linked with self-reported LTPA, but only planning was linked with device-assessed LTPA. Within-person level results are in line with theories of intrapersonal behaviour change (e.g., HAPA; Schwarzer, 2008) and previous studies demonstrating positive links between self-efficacy, and planning, and PA (Carraro & Gaudreau, 2013; Zhang et al., 2019). Our findings further indicate that these processes manifest on a daily time scale (Anderson, 2021; Pickering et al., 2016). Methodological issues, such as larger within- than between-person variability, could help explain why self-efficacy was related to device-assessed LTPA at the within-person, but not the between-person level. Results for self-reported LTPA may have been less affected, due to stronger associations between self-efficacy and self-reported LTPA – two measures which underlie subjective judgements.

Unexpectedly, positive affect did not emerge as a meaningful LTPA predictor at the within- or between-person level. This finding contrasts with prior research (Emerson et al., 2018; Niermann et al., 2016), possibly because affect was not measured in close temporal proximity to activities, unlike in Niermann et al. (2016).

### Interplay between intrapersonal processes and social support

Within-person mediation analyses revealed that the effects of self-efficacy and planning on LTPA were mediated by social support from family/friends. Social support mediated 16%−22% of the positive daily relationships between self-efficacy/planning and LTPA, both device-assessed and self-reported. Our findings are in line with previous research suggesting that self-efficacy and active coping, as indicated by planning, are recipient characteristics that elicit or link to support provision (Iida et al., 2008; Knoll et al., 2011; Schwaninger et al., 2021; Schwarzer & Weiner, 1991). If individuals are self-efficacious and plan their LTPA, they are more likely to receive LTPA-specific support. These within-person mediation results also complement between-person level mediational findings in previous studies (Banik et al., 2021; Hohl et al., 2016). A phenomenon frequently discussed in sociology, the ‘Matthew effect’ describes that those with a higher level of resources accumulate more advantages over time compared to those with fewer resources whose disadvantage increases (‘the rich get richer’; DiPrete & Eirich, 2006, p. 2). Our findings suggest that the Matthew effect is also evident in the area of social exchange processes: Those who have more intrapersonal resources (such as self-efficacy, planning) receive more support than those with fewer intrapersonal resources. Future studies should explore positive feedback loops between self-efficacy or planning and social support in promoting LTPA at a within-person level. Moreover, interventions to foster social support and disrupt this mechanism for individuals with fewer resources could be designed.

### Strengths and limitations

Our study has several major strengths, including the collection of intensive-longitudinal data, which reduces recall bias (Shiffman et al., 2008) and allows to accommodate individual differences in the highly heterogeneous processes of social support (Hamaker, 2025). Another strength was the use of two measures of LTPA: Device-assessed LTPA avoids the overestimation bias often encountered in PA self-reports, while self-reported LTPA has the ability to capture specific types of LTPA (e.g., water-based activities, for which our accelerometers cannot be worn). The two measures corresponded moderately-to-strongly (*r*_within-person_ = .50, *r*_between-person_ = .47), exceeding the moderate associations found in prior studies (Cerin et al., 2016), supporting the validity of our AA self-reported LTPA measurement. In terms of limitations, not including light-intensity physical activity in our device-assessed MVPA LTPA measure may have overlooked relevant health-promoting activities, e.g. of muscle-strengthening type, that cannot be captured well by accelerometers. Although our self-reported work-/leisure-times may be subject to biases (e.g., overestimation), and could therefore affect our device-assessed LTPA measure, combining daily self-reported worktime logs with high-resolution movement data provides a viable assessment approach (Hylkema et al., 2021; Ketels et al., 2019). Except for positive affect, we used single-item indicators, primarily to increase adherence and reduce participant burden. Single-item measures of fluctuating cognitive outcomes impede traditional reliability testing approaches, leaving reliability unknown (Allen et al., 2022), despite our efforts to validate them against multi-item scales. Song et al. (2022) compared single-item and multiple-item scales in an intensive-longitudinal study and found evidence of concurrent and predictive validity, and only minor advantages of multiple-item scales.

We did not assess relationship characteristics, or differentiate between relationship types within domains, limiting interpretation across social support sources. Current knowledge about non-romantic dyads remains limited. Future studies should specify support providers for each assessment, and include relationship quality and relationship commitment measures, and also explore under which conditions momentary support from the work domain may have positive effects on leisure-time activities. Our study focused exclusively on the recipients' perspective. To gain a more nuanced view of social exchanges, future studies should incorporate the perspective of the support provider through dyadic designs (Hohl et al., 2016). Given the high variability of effects between participants, future studies may also employ micro-randomized experimental designs (Klasnja et al., 2015) to make within-subject comparisons and to test the time-varying effect of social support across different kinds of dyads.

While accounting for time and other covariates, our observational, intensive-longitudinal design precludes causal or time-order inference. Several alternative directions of effects are possible. One plausible alternative order is the *enabling hypothesis* which proposes that social support promotes health-related outcomes by facilitating intrapersonal processes such as self-efficacy (Banik et al., 2021; Benight & Bandura, 2004; Schwarzer & Knoll, 2007). Due to our measurement design with within-day ordered assessments of intraindividual self-efficacy, planning, and positive affect (morning) and social exchange processes (afternoon), we could not test the enabling hypothesis without stretching the analyses to effects across days (i.e., using previous-day social support), obliterating the crucial time resolution of a single day (Scholz et al., 2021). To our knowledge, this is the first study to examine the cultivation hypothesis within a single day, incorporating temporal precedence of predictor variables.

### Implications

Our findings on the within-person associations of private-domain social support, self-efficacy, and planning with LTPA, mark these processes as potential intervention targets for ecological momentary interventions to promote LTPA, e.g., for Just-In-Time Adaptive Interventions (Nahum-Shani et al., 2018). Providing interventions at times when a person needs them could help to overcome daily barriers towards LTPA. Our findings supporting the cultivation hypothesis suggest that fostering self-efficacy and planning in interventions might be worthwhile. Both appear to boost social support from the private domain.

## Conclusions

We found that social support from family/friends, self-efficacy, and planning are relevant day-to-day processes linked to higher levels of LTPA. These findings support the cultivation hypothesis – a mediating role of social support in the association between self-efficacy and planning, and LTPA – and highlight promising targets for future interventions.

## Supplementary Material

Supplementary material Supplementary Material

## Data Availability

The data that support the findings of this study are available from the authors upon reasonable request.
