# Supplementary material for: Daily self-efficacy, planning and social support explain leisure-time physical activity in working adults: evidence for the cultivation hypothesis from an ambulatory assessment study
Source: Health Psychol Behav Med. 2025 Nov 12;13(1):2576610. doi: 10.1080/21642850.2025.2576610 (PMC12613304; doi:10.1080/21642850.2025.2576610)
Supplement: Supplementary material [file RHPB_A_2576610_SM8421.docx]

Supplementary Material

**Daily self-efficacy, planning and social support explain leisure-time physical activity in working adults: Evidence for the cultivation hypothesis from an ambulatory assessment study**

Wilhelm, L. O., Knoll, N., Diering, L.-E., Kolodziejczak-Krupp, K., Maas, J., Schmidt, H., & Fleig, L.

Overview

| Supplementary Material S1 | Figure S1. Data Availability Flowchart. | …………………….3 |
| --- | --- | --- |
| Supplementary Material S2 | Table S2*. Parameter Estimates from Mixed Model Testing Daily Social Support from Colleagues/Supervisors and from Family/Friends (Model A), and Daily Social Support from Family/Friends, Positive Affect, Self-efficacy, and Planning (Model B) and their Link with Daily Leisure-time Physical Activity (self-reported LTPA/day, Winsorized 90^th^ Percentile)* | …………………….5 |
| Supplementary Material S3 | Table S3*. Parameter Estimates from Fully-Controlled Mixed Model Testing Daily Social Support from Colleagues/Supervisors and from Family/Friends (Model A), and Daily Social Support from Family/Friends, Positive Affect, Self-Efficacy, and Planning (Model B) and their Link with Daily Leisure-time Physical Activity (device-assessed MVPA during leisure, Winsorized 90^th^ Percentile)* | …………………….7 |
| Supplementary Material S4 | Table S4*. Summary of Within-person Mediation Analyses of Self-Efficacy, or Planning, and Their Effects on Leisure-time Physical Activity (device-assessed MVPA/day and self-reported LTPA/day, Winsorized 90^th^ Percentile) via Increased Leisure-time specific Received Social Support from Family/Friends* | …………………….10 |
|  |  |  |

# Supplementary Material S1

## Figure S1

*Data Availability Flowchart*
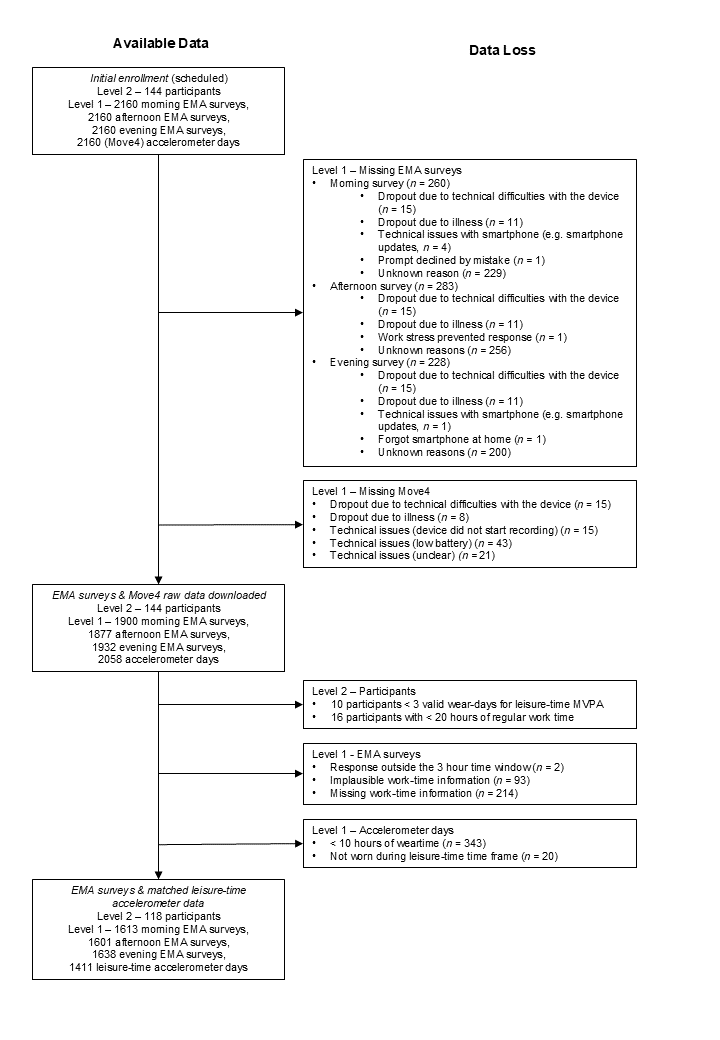


*Note.* Level 1 represents days, Level 2 represents participants. Flowchart adapted from Maher et al. (2018). EMA = Ecological momentary assessment, MVPA = moderate-to-vigorous physical activity. Reference: Maher, J. P., Rebar, A. L., & Dunton, G. F. (2018). Ecological momentary assessment is a feasible and valid methodological tool to measure older adults’ physical activity and sedentary behavior. *Frontiers in Psychology*, *9*(1485). <https://doi.org/10.3389/fpsyg.2018.01485>

# Supplementary Material S2

## Table S2

*Parameter Estimates from Mixed Model Testing Daily Social Support from Colleagues/Supervisors and from Family/Friends (Model A), and Daily Social Support from Family/Friends, Positive Affect, Self-efficacy, and Planning (Model B) and their Link with Daily Leisure-time Physical Activity (self-reported LTPA/day, Winsorized 90^th^ Percentile)*

|  | *Model A* | | | | *Model B* | | | |
| --- | --- | --- | --- | --- | --- | --- | --- | --- |
| Fixed effects | *Logs (SE)* | *IRR* | 95% CI | *p* | *Logs (SE)* | *IRR* | 95% CI | *p* |
| Intercept | 3.94 (0.21) | 51.34 | 3.53 – 4.35 | **<.001** | 3.47 (0.20) | 32.29 | 3.07 – 3.88 | **<.001** |
| Linear time^a^ (reference = day 1) | 0.001 (0.01) | 1.00 | -0.03 – 0.03 | .936 | 0.01 (0.01) | 1.01 | -0.02 – 0.03 | .618 |
| Working day | -0.39 (0.16) | 0.67 | -0.71 – -0.08 | **.015** | -0.29 (0.16) | 0.75 | -0.60 – 0.03 | .072 |
| Weekend | -0.05 (0.18) | 0.95 | -0.40 – 0.31 | .795 | 0.26 (0.18) | 1.29 | -0.09 – 0.61 | .148 |
| Within-person effects |  |  |  |  |  |  |  |  |
| Social support from colleagues/supervisor | 0.06 (0.07) | 1.06 | -0.07 – 0.19 | .355 |  |  |  |  |
| Social support from family/friends | 0.24 (0.05) | 1.27 | 0.14 – 0.34 | **<.001** | 0.17 (0.05) | 1.18 | 0.07 – 0.27 | **.001** |
| Positive affect |  |  |  |  | 0.02 (0.10) | 1.02 | -0.18 – 0.22 | .847 |
| Self-efficacy |  |  |  |  | 0.23 (0.06) | 1.26 | 0.11 – 0.35 | **<.001** |
| Planning |  |  |  |  | 0.14 (0.06) | 1.15 | 0.03 – 0.25 | **.012** |
| Between-person effects |  |  |  |  |  |  |  |  |
| Social support from colleagues/supervisor | -0.15 (0.13) | 0.86 | -0.40 – 0.10 | .251 |  |  |  |  |
| Social support from family/friends | 0.22 (0.08) | 1.24 | 0.06 – 0.37 | **.005** | 0.09 (0.06) | 1.10 | -0.02 – 0.21 | .119 |
| Positive affect |  |  |  |  | 0.07 (0.13) | 1.07 | -0.19 – 0.33 | .608 |
| Self-efficacy |  |  |  |  | 0.21 (0.09) | 1.23 | 0.04 – 0.38 | **.018** |
| Planning |  |  |  |  | 0.28 (0.09) | 1.32 | 0.11 – 0.45 | **.001** |
| Age | 0.01 (0.01) | 1.01 | -0.01 – 0.02 | .283 | <0.001 (0.01) | 1.00 | -0.01 – 0.01 | .883 |
| Gender: man (reference = woman) | 0.15 (0.15) | 1.17 | -0.14 – 0.45 | .302 | 0.27 (0.15) | 1.30 | -0.03 – 0.56 | .077 |
| Occupational context: Sedentary (reference = physiotherapist) | -0.40 (0.18) | 0.67 | -0.76 – -0.05 | **.027** | -0.18 (0.14) | 0.83 | -0.46 – 0.10 | .206 |
| Being single | 0.17 (0.17) | 1.18 | -0.16 – 0.50 | .320 | 0.10 (0.16) | 1.10 | -0.21 – 0.41 | .527 |
| Random effects ([co-]variances) | Var | | 95% CI | | Var | | 95% CI | |
| *Level-2 (between-person)* |  | |  | |  | |  | |
| Intercept | 0.11 | | 0.01 – 0.85 | | 0.01 | | <.001 – 932.24 | |
| *Within-person effects* |  | |  | |  | |  | |
| Social support from colleagues/supervisors | <0.001 | | <0.001 – <0.001 | | - | | - | |
| Social support from family/friends | <0.001 | | <0.001 – <0.001 | | <0.001 | | <0.001 – <0.001 | |
| Positive affect | - | |  | | <0.001 | | <0.001 – <0.001 | |
| Self-efficacy | - | |  | | <0.001 | | <0.001 – <0.001 | |
| Planning | - | |  | | <0.001 | | <0.001 – <0.001 | |
| Model fit |  | | | |  | | | |
| Residual Dispersion | 0.20 | | | | 0.22 | | | |
| Deviance | 1426.33 | | | | 1322.473 | | | |
| AIC/BIC | 11382.18/11466.84 | | | | 10294.88/10409.09 | | | |

*Note.* 1468 observations, 118 individuals (Model A), 1351 observations, 118 individuals (Model B). LTPA = leisure-time moderate-to-vigorous physical activity; Logs = Odds ratio; IRR = Incidence rate ratio, i.e. exponentiated estimates; *SE* = Standard Error; CI = confidence interval with lower level and upper level; Var = Variance; AIC = Akaike information criterion; BIC = Bayesian information criterion. Significant *p*-values are printed in bold. Due to model non-convergence, no random effects were specified for linear time, and no correlations of random effects.

# Supplementary Material S3

## Table S3

*Parameter Estimates from Fully-Controlled Mixed Model Testing Daily Social Support from Colleagues/Supervisors and from Family/Friends (Model A), and Daily Social Support from Family/Friends, Positive Affect, Self-Efficacy, and Planning (Model B) and their Link with Daily Leisure-time Physical Activity (device-assessed MVPA during leisure, Winsorized 90^th^ Percentile)*

|  | *Model A* | | | *Model B* | | |  |
| --- | --- | --- | --- | --- | --- | --- | --- |
| Fixed effects | *B (SE)* | 95% CI | *p* | *B (SE)* | 95% CI | *p* |  |
| Intercept | 63.05 (6.28) | 50.72 – 75.38 | **<.001** | 58.23 (6.32) | 45.84 – 70.63 | **<.001** |  |
| Linear time^a^ (reference = day 1) | 0.03 (0.18) | -0.33 – 0.39 | .851 | 0.02 (0.18) | -0.34 – 0.38 | .909 |  |
| Working day | -1.77 (3.21) | -8.08 – 4.53 | .581 | -0.49 (3.18) | -6.73 – 5.75 | .876 |  |
| Weartime during leisure (in hours) | 3.49 (0.38) | 2.74 – 4.23 | **<.001** | 3.57 (0.38) | 2.83 – 4.32 | **<.001** |  |
| Weekend | -7.70 (2.58) | -12.76 – -2.64 | **.003** | -5.31 (2.56) | -10.34 – -0.28 | **.039** |  |
| Within-person effects |  |  |  |  |  |  |  |
| Social support from colleagues/supervisors | 0.36 (1.12) | -1.84 – 2.57 | .747 | - | - | - |  |
| Social support from family/friends | 5.70 (0.78) | 4.17 – 7.23 | **<.001** | 3.85 (0.75) | 2.38 – 5.32 | **<.001** |  |
| Positive affect |  |  |  | 3.03 (1.69) | -0.29 – 6.35 | .073 |  |
| Self-efficacy |  |  |  | 2.50 (0.85) | 0.84 – 4.16 | **.003** |  |
| Planning |  |  |  | 2.33 (0.75) | 0.85 – 3.81 | **.002** |  |
| Between-person effects |  |  |  |  |  |  |  |
| Social support from colleagues/supervisors | 8.60 (3.35) | 1.96 – 15.24 | **.012** |  |  |  |  |
| Social support from family/friends | 1.10 (2.24) | -3.35 – 5.55 | .626 | 2.15 (2.08) | -1.97 – 6.27 | .302 |  |
| Positive affect |  |  |  | -1.16 (4.02) | -9.14 – 6.83 | .774 |  |
| Self-efficacy |  |  |  | -1.19 (3.11) | -7.37 – 4.99 | .704 |  |
| Planning |  |  |  | 10.18 (3.11) | 4.00 – 16.36 | **.001** |  |
| Age | 0.13 (0.24) | -0.34 – 0.61 | .583 | -0.13 (0.24) | -0.61 – 0.35 | .583 |  |
| Gender: man (reference = woman) | -3.18 (4.79) | -12.69 – 6.33 | .508 | 3.66 (4.69) | -5.65 – 12.97 | .437 |  |
| Occupational context: Sedentary (reference = physiotherapist) | 2.82 (4.55) | -6.21 – 11.84 | .537 | -1.07 (4.94) | -10.87 – 8.73 | .828 |  |
| Being single | 6.84 (5.98) | -5.02 – 18.69 | .255 | 12.58 (6.03) | 0.60 – 24.55 | **.040** |  |
| Living with partner | -5.10 (5.23) | -15.47 – 5.28 | .332 | -1.24 (5.32) | -11.80 – 9.32 | .816 |  |
| Having kids | 0.48 (5.30) | -10.05 – 11.00 | .928 | -1.51 (5.38) | -12.18 – 9.17 | .780 |  |
| Body mass index | -0.47 (0.72) | -1.90 – 0.97 | .520 | -0.83 (0.74) | -2.30 – 0.65 | .269 |  |
| Average weekly working hours | 0.20 (0.33) | -0.46 – 0.86 | .546 | 0.32 (0.35) | -0.37 – 1.01 | .362 |  |
| Studying | 3.00 (8.37) | -13.61 – 19.60 | .721 | 2.10 (8.37) | -14.52 – 18.72 | .802 |  |
| Low income | 8.06 (10.07) | -11.91 – 28.04 | .425 | -2.63 (10.11) | -22.69 – 17.43 | .795 |  |
| Primarily working from home | -3.50 (6.12) | -15.63 – 8.64 | .569 | -2.89 (6.15) | -15.10 – 9.33 | .640 |  |
| Random effects ([co-]variances) | Var | 95% CI | | Var | 95% CI | |  |
| *Level-2 (between-person)* |  |  | |  |  | |  |
| Intercept | 394.51 | 284.81 – 546.48 | | 371.89 | 268.32 – 515.44 | |  |
| *Within-person effects* |  |  | |  |  | |  |
| Social support from colleagues/supervisors | 21.61 | 7.00 – 66.68 | | - | - | |  |
| Social support from family/friends | 16.92 | 7.52 – 38.10 | | 11.64 | 3.98 – 34.06 | |  |
| Positive affect | - | - | | 79.54 | 29.88 – 211.79 | |  |
| Self-efficacy | - | - | | 1.03 | 0.29 – 3.61 | |  |
| Planning | - | - | | 5.87 | 1.31 – 26.26 | |  |
| Intercept and social support from colleagues/supervisors | -.08 | -0.63 – 0.51 | | - | - | |  |
| Intercept and social support from family/friends | .51 | -0.004 – 0.81 | | - | - | |  |
| Social support from colleagues/supervisors and from family/friends | -.82 | -0.99 – 0.54 | | - | - | |  |
| *Level-1 (within-person)* |  |  | |  |  | |  |
| Residuals | 697.79 | 640.23 – 760.53 | | 609.86 | 554.05 – 671.29 | |  |
| Model fit |  | | |  | | |  |
| Deviance | 11978.76 | | | 11120.3 | | |  |
| AIC/BIC | 11970.46/12108.56 | | | 11110.77/11262.11 | | |  |
| *R*² (marginal/conditional) | .22/.52 | | | .29/.59 | | |  |

*Note.* 1250 observations, 113 individuals (Model A), 1171 observations, 113 individuals (Model B). LTPA = leisure-time moderate-to-vigorous physical activity; SE = Standard Error; CI = confidence interval with lower level and upper level; AIC = Akaike information criterion; BIC = Bayesian information criterion. Unstandardized coefficients are displayed. Significant *p*-values are printed in bold. Due to model non-convergence, no random effects were specified for linear time. In Model B, due to non-positive definiteness, a variance components random effect variance-covariance structure was specified (i.e., covariances constrained to zero).

# Supplementary Material S4

## Table S4

*Summary of Within-person Mediation Analyses of Self-Efficacy, or Planning, and Their Effects on Leisure-time Physical Activity (device-assessed MVPA during leisure-time/day and self-reported LTPA/day, Winsorized 90^th^ Percentile) via Increased Leisure-time specific Received Social Support from Family/Friends*

| Independent variable (X) | Mediator (M) | Dependent variable (Y) | Effect of X on M (a) | | Effect of M on Y (b) | | Direct effect (c’) | | Indirect effect  (a b) | | Total effect (c) | | Ratio indirect effect / total effect |
| --- | --- | --- | --- | --- | --- | --- | --- | --- | --- | --- | --- | --- | --- |
|  |  |  | *Est (SE)* | *p* | *Est (SE)* | *p* | *Est (SE)* | *p* | *Est (SE)* | *90% CI* | *Est (SE)* | *p* |  |
| *Dependent variable: Device-assessed LTPA* | | | | | | | | | | | | | |
| Self-efficacy | Social support from family/friends | Device-assessed LTPA | 0.26 (0.04) | <.001 | 3.80 (0.78) | <.001 | 4.35 (0.78) | <.001 | 0.90 (0.38) | [0.27; 1.52] | 5.25 (0.77) | <.001 | 17% |
| Planning | Social support from family/friends | Device-assessed LTPA | 0.26 (0.04) | <.001 | 3.23 (0.74) | <.001 | 4.10 (0.66) | <.001 | 0.80 (0.33) | [0.26; 1.34] | 4.90 (0.68) | <.001 | 16% |
| *Dependent variable: Self-reported LTPA* | | | | | | | | | | | | | |
| Self-efficacy | Social support from family/friends | Self-reported LTPA | 0.28 (0.04) | <.001 | 7.07 (1.22) | <.001 | 9.45 (1.08) | <.001 | 2.31 (0.65) | [1.24, 3.38] | 11.76 (1.09) | <.001 | 20% |
| Planning | Social support from family/friends | Self-reported LTPA | 0.27 (0.04) | <.001 | 7.02 (1.14) | <.001 | 8.27 (0.83) | <.001 | 2.31 (0.54) | [1.43, 3.19] | 10.58 (0.85) | <.001 | 22% |

*Note.* 1120 ≤ *n* ≤ 1231 observations, 108 participants (due to missings, or lack of within-person variation). X = Independent variable (X1, X2); M = Mediating variable; Y = Dependent variable; Est = Estimate; SE = Standard Error; CI = Confidence Interval; LTPA = Leisure-time physical activity. Covariates (Level-1): linear time trend, working day, weekend, (device-assessed LTPA as Y) leisure-time device-weartime (in hours).
